# HOW NOT TO DATA ARCHIVE IN 10 WAYS

**DOI:** 10.1093/geroni/igad104.2506

**Published:** 2023-12-21

**Authors:** Ashley Millenbah, Elizabeth Albers, Katie Louwagie

**Affiliations:** University of Minnesota, Minneapolis, Minnesota, United States; University of Minnesota School of Public Health, Minneapolis, Minnesota, United States; University of Minnesota, Minneapolis, Minnesota, United States

## Abstract

Data sharing offers opportunities to replicate published findings, improves transparency in science, and increases the impact of the research by allowing other researchers to conduct new analyses. Effective January 25, 2023, the National Institute on Aging has new mandates for data sharing. However, many researchers are not experienced in data sharing and curation. Researchers will need to become more familiar with data sharing, specifically archiving data in data repositories. With these new requirements, many teams are attempting data sharing or archiving for the first time. We present 10 lessons learned the hard way, from real experience, prohibiting successful data archiving. Many of the challenges encountered could be avoided by effective planning, such as securing funding for curation and/or de-identification, adding data archiving details into the grant, and finding multiple repositories to potentially use. Other problems can be avoided by writing a consent form that allows for data archiving, such as by not limiting access to data, or limiting the type or format of data to be shared. Consulting with a legal team with experience in data archiving, such as at a data repository, is also recommended. By avoiding these pitfalls, researchers can successfully archive their data, ensuring their data are maintained and usable for other researchers in the future.

